# Use of Anticoagulant Therapy in Patients with Acute Myocardial Infarction and Atrial Fibrillation

**DOI:** 10.3390/medicina58030338

**Published:** 2022-02-23

**Authors:** Ratko Lasica, Lazar Djukanovic, Dejana Popovic, Lidija Savic, Igor Mrdovic, Nebojsa Radovanovic, Mina Radosavljevic Radovanovic, Marija Polovina, Radan Stojanovic, Dragan Matic, Ana Uscumlic, Milika Asanin

**Affiliations:** 1 Department of Cardiology, Emergency Center-Clinical Center of Serbia, 11000 Belgrade, Serbia; lazardjukanovic08@gmail.com (L.D.); lidijasavic2007@gmail.com (L.S.); igormrd@gmail.com (I.M.); radovanovicn@sbb.rs (N.R.); marija_polovina@yahoo.com (M.P.); anauscumlic@gmail.com (A.U.); masanin2013@gmail.com (M.A.); 2 Department of Cardiology, Clinical Center of Serbia, 11000 Belgrade, Serbia; dejanapopovic@yahoo.co.uk (D.P.); mivasrce14@gmail.com (M.R.R.); dragan4m@gmail.com (D.M.); 3 Department of Pharmacology, Clinical Pharmacology and Toxicology, Faculty of Medicine, University of Belgrade, 11000 Belgrade, Serbia; radan@doctor.com

**Keywords:** acute myocardial infarction, atrial fibrillation, anticoagulant therapy

## Abstract

The incidence of atrial fibrillation (AF) in acute coronary syndrome (ACS) ranges from 2.3–23%. This difference in the incidence of AF is explained by the different ages of the patients in different studies and the different times of application of both reperfusion and drug therapies in acute myocardial infarction (AMI). About 6–8% of patients who underwent percutaneous intervention within AMI have an indication for oral anticoagulant therapy with vitamin K antagonists or new oral anticoagulants (NOAC).The use of oral anticoagulant therapy should be consistent with individual risk of bleeding as well as ischemic risk. Both HAS-BLED and CHA2DS2VASc scores are most commonly used for risk assessment. Except in patients with mechanical valves and antiphospholipid syndrome, NOACs have an advantage over vitamin K antagonists (VKAs). One of the advantages of NOACs is the use of fixed doses, where there is no need for successive INR controls, which increases the patient’s compliance in taking these drugs. The use of triple therapy in ACS is indicated in the case of patients with AF, mechanical valves as well as venous thromboembolism. The results of the studies showed that when choosing a P2Y12 receptor blocker, less potent P2Y12 blockers such as Clopidogrel should be chosen, due to the lower risk of bleeding. It has been proven that the presence of AF within AMI is associated with a higher degree of reinfarction, more frequent stroke, high incidence of heart failure, and there is a correlation with an increased risk of sudden cardiac death. With the appearance of AF in ACS, its rapid conversion into sinus rhythm is necessary, and in the last resort, good control of heart rate in order to avoid the occurrence of adverse clinical events.

## 1. Introduction

Atrial fibrillation is an atrial tachyarrhythmia characterized by uncoordinated atrial depolarization, with impaired mechanical function and variable, irregular ventricular frequency. In the world, AF is the most common long-term cardiac arrhythmia in the adult population with an estimated prevalence of 2–4%. An increase in AF in the future is predicted due to extended life expectancy as well as more intensive searches for asymptomatic AF in the population of patients at risk [1].

According to the World Health Organization, cardiovascular diseases are the leading cause of morbidity and mortality in the world, of which AMI is the most common. Acute coronary syndrome is the cause of more than 4 million deaths in Europe and Asia, as well as more than 2.4 million deaths in the United States each year. Although mortality from ACS is still high, it has been declining in recent decades, both due to lifestyle changes in primary prevention and the use of modern evidence-based therapy. ACS, which is accompanied by complications such as heart failure or the appearance of malignant arrhythmias, is associated with a worse prognosis. It is estimated that approximately 90% of patients with ACS have some of the arrhythmias in the first 24 h after the onset of the disease. The occurrence of AF during ACS is associated with a longer period of hospitalization, frequent complications and higher both in-hospital and overall mortality [2].

The incidence of AF in acute coronary syndrome, according to studies, ranges between 2.3–23% [1,3]. This difference in the incidence of AF is explained by the different ages of the patients within different studies and the different times of application of both reperfusion and drug therapies in AMI. Randomized TRACE and OPTIMAAL studies examining the efficacy of drugs such as ACE inhibitors or ARBs in ACS have shown a lower incidence of AF in the ACS framework, which could be explained by the antiarrhythmic effect of these drugs [4,5].

In addition to the fact that ACS poses a risk for AF, the REGARDS study showed that AF increases the risk of developing ACS more than 2 times, especially in the female population [6]. Similar results were shown by the ARIC study, although the association of AF with the occurrence of ACS has been demonstrated only for the subpopulation of patients who had AMI without ST-segment elevation (NSTEMI) [7]. The literature lists some of the mechanisms for the occurrence of AF within AMI: atrial ischemia, sinoatrial node ischemia, right ventricular ischemia, and left ventricular dysfunction, acute heart failure leading to atrial fibrillation and thus stimulating cardiomyocyte excitability, sympathetic dysfunction, endothelial dysfunction and systemic inflammation.

The effect of AF on AMI occurrence is explained by the induction of coronary thromboembolism, which accounts for approximately 3% of all AMI [8]. Another reason may be the reduced blood supply to the coronary blood vessels due to the tachyarrhythmic effect on the shortening of diastole. In addition, the influence of inflammation in both AMI and AF affects the development and progression of coronary heart disease.

## 2. Association of Atrial Fibrillation and AMI

Atrial fibrillation is one of the most common arrhythmias in patients with AMI. Among the first studies to examine the frequency and prognostic significance of AF within NSTEMI and ST-elevation myocardial infarction (STEMI) was the RICO study. This study did not show a significant difference in the frequency of AF between patients with AMI with or without ST elevation [9]. In this study, AF was shown to be an independent predictor of death in patients with NSTEMI. Similar results were presented in a retrospective cohort study performed on 6705 patients, of which 3094 patients had STEMI while 3611 had NSTEMI.

Atrial fibrillation was registered in 5.4% of patients, with the incidence of AF in patients with STEMI and NSTEMI being similar (5% vs. 5.6%, respectively); in 2.1% of patients AF was diagnosed earlier, while it was detected as new in 3.2% of patients [10]. Compared to previous studies that used a standard electrocardiogram to diagnose arrhythmias in patients with AMI, the CHARISMA study was among the first studies to use continuous heart rate monitoring to detect arrhythmias within AMI in patients with reduced ejection fraction (EF).

In this study, the incidence of newly developed AF was estimated at 28% over a 1.9-year follow-up period [11]. Analysis of AREST study data concluded that the cumulative incidence of AF after AMI in patients with preserved EF is quite underestimated due to numerous asymptomatic cases (approximately 90%) and the inability to detect them without a continuous electrocardiogram. This study showed that more than 60% of patients after AMI have newly developed AF in the 24-month follow-up period, with the highest incidence between 3 and 6 months of follow-up [12].

The discrepancy between the AF frequencies within these two studies can be explained by the earlier start of rhythm monitoring within the AREST study as well as by the application of a more accurate algorithm for AF detection. Recent studies (SCREEN-AF and The LOOP study) that examined, among other primary goals, the incidence of AF in the general population independent of ACS found that the use of continuous monitoring to detect arrhythmias greatly increased the detection of asymptomatic cases of AF. The importance of paroxysms of asymptomatic AF as well as the need for their treatment is still debatable [13,14].

## 3. Association of ACS Treatment with the Incidence of AF

The use of drug reperfusion therapy (thrombolytic therapy) in AMI has reduced both mortality and the incidence of major adverse cardiac events (MACE). Nielsen et al. showed in a small number of patients a decrease in the incidence of AF in patients who received thrombolytic therapy compared to those treated conservatively [15]. Eldar et al. confirmed this opinion by comparing patients who received thrombolytic therapy (46.3%) to those who did not receive thrombolytic therapy. A significantly lower incidence of paroxysmal AF was registered in patients treated with thrombolytic therapy (6.7% vs. 10.8%).

Additionally, in his study, comparing the occurrence of AF in the period before thrombolytic therapy with the period of thrombolytic therapy, a similar incidence of paroxysmal AF was observed (9.9% vs. 8.9%). Patients with AF in the thrombolytic era have been shown to be older, with a higher incidence of comorbidities compared with patients in the prethrombolytic era [16].

Between 1990 and 1997, Goldberg et al. published a study showing a decline in the incidence of AF from 18% to 11% in patients after AMI [17]. An incidence of AF of 10.4% was registered in the GUSTO I study among patients with AMI treated with fibrinolytic therapy [18]. Among the first studies to assess the incidence of AF in patients with acute coronary syndrome who underwent PCI within 24 h of symptoms onset, one showed an incidence of AF of 12%, with 4.3% of patients having AF on admission and 7.7% developing AF during hospitalization [19]. Moreover, Podolecki et al. published a study conducted in the period from 2003 to 2008, which included 2980 patients with AMI who were treated with an invasive approach. Atrial fibrillation was registered in 9.5% of patients [20].

## 4. Presence of Risk Factors for AF in AMI

**Figure 1:**
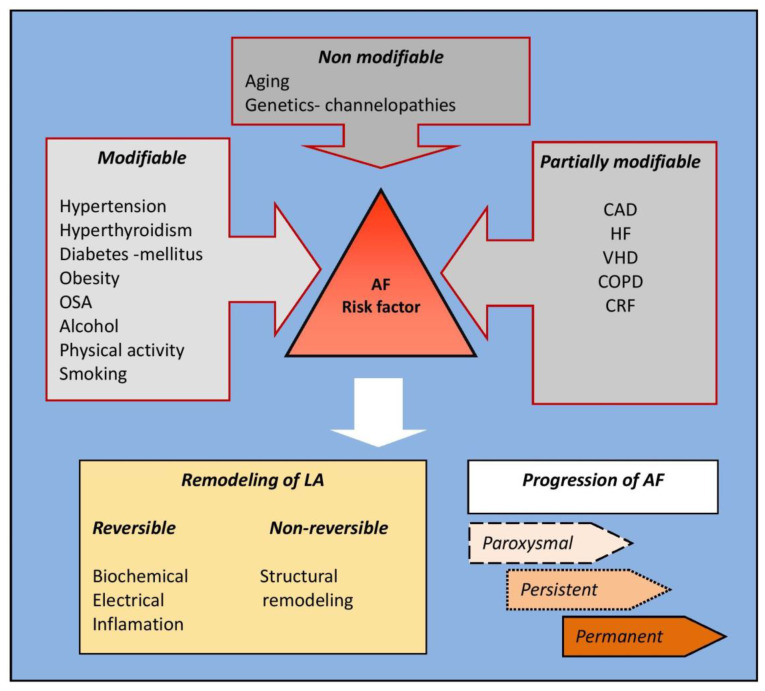
Cardiovascular risk factors for the development of AF [1]: Legend: AF—atrial fibrillation; CAD—coronary artery disease; COPD—chronic obstructive pulmonary disease; LA—left antrum; OSA—obstructive sleep apnea; HF—heart failure; VHD—valvular heart disease; CRF—chronic renal failure.

Atrial fibrillation is a common arrhythmia in patients with ACS, and its occurrence is more frequent if risk factors are present (Figure 1).

## 5. AF Predictors in AMI

Previous studies have shown that changes in the values of some laboratory parameters, pathological findings of echocardiography and the occurrence of heart failure during AMI may be associated with the occurrence of AF.

### 5.1. Laboratory Parameters

Within ACS, the most frequently examined predictors of AF were N-terminal prohormone of brain natriuretic peptide (NT-pro BNP), C reactive protein (CRP) and Troponin, which are routinely used as diagnostic and prognostic laboratory parameters in patients with ACS. According to the TRIUMPH registry, independent predictors of AF development in AMI were NT-pro BNP as well as CRP [21]. A Dorje et al. study conducted in the period from January 2008 to December 2011 included 268 patients with AMI. New AF was registered in 13.4% of respondents.

After multivariate analysis, the B-type NP value ≥ 796 pg/mL was an independent predictor of newly developed AF in these patients (OR 5.1, 95% CI 1.7–15.5, *p* = 0.004) [22]. Asanin et al., in a study of patients with STEMI treated with primary PCI, showed that patients with elevated B-type natriuretic protein 24 h after the onset of symptoms had a higher incidence of AF than the control group [23]. The predictive values of BNP for the occurrence of AF is proven in the general population although not in all studies [24,25]. As recent research supports the association of systemic inflammation with the development and maintenance of AF, as well as the importance of the inflammatory response to tissue damage within AMI, the predictive value of CRP as an independent indicator of systemic inflammation was considered.

In a study by Aronson et al. that included patients with AMI, after a multivariate analysis CRP proved to be an independent predictor of this arrhythmia [26]. Finally, Troponin T biomarker specific for myocardial damage has not been shown, in both the TRIUMPH study and other studies, to be an independent predictor of AF development within AMI [23]. The results of Gal et al. showed an association between hsTnT values measured at different intervals and newly developed AF between 24–72 h as well as 72 h after hospital admission [27]. Such data are also supported by a recent study by Raczkowska-Golanko et al. [28].

It should always be borne in mind that elevated levels of Troponin may be associated with a variety of cardiac and non-cardiac conditions. Cardiac conditions with increased Troponin values are: acute and chronic heart failure, acute myocarditis, arrhythmias and aortic dissections, while non-cardiac conditions are pulmonary embolism, sepsis, stroke and many others [29,30]. Troponin levels are also elevated in patients with renal insufficiency and during strenuous exercise [31,32].

### 5.2. Echocardiographic Parameters

It has long been known that the likelihood of developing AF within the general population increases with increasing left atrial diameter as well as decreasing left ventricular ejection fraction (LVEF) [33]. According to a meta-analysis that included ten studies with patients with newly diagnosed AF, decreased LVEF as well as increased left atrial diameter were associated with a higher risk of AF after AMI [34]. In addition, according to a study by Raczkowska-Golanko et al., left atrial diameter ≥ 41 mm and LVEF ≤ 44% were significant predictors of newly developed AF in univariate analysis in patients with AMI. EF has also been shown to be significant in multivariate analysis [28].

### 5.3. Heart Failure in AMI as a Predictor of AF Development

Heart failure is the most significant predictor of AF development in patients with AMI. In a study by Asanin et al., heart failure within the AMI was examined as an independent predictor of AF. New AF was classified as early if it occurred within 24 h of the onset of heart failure symptoms and late if it occurred after this period. Heart failure according to multivariate analysis was the most important predictor of AF (OR 3.03, 95% CI, 2.08–4.43, *p* < 0.0001) [35]. Moreover, in patients with AMI, the presence of advanced diastolic dysfunction was independently associated with new-onset AF [36,37].

## 6. Influence of AF on Clinical Outcome within AMI

Atrial fibrillation is associated with significant morbidity and mortality, and poses a significant burden to patients, public health and the health economy. According to the ESC Guidelines for 2021, AF is independently associated with twice the mortality in the female population and 1.5 times the mortality in the male population [1]. The GUSTO I study showed that newly developed AF was an independent predictor of both 30-day and 1-year mortality in patients with ACS. Patients who develop AF during hospitalization are more likely to have heart failure, reinfarction and asystole [18]. In the GISSI-3 study, which included patients with ACS, it was shown that in the group of patients who developed AF, the intrahospital mortality was 12.6%, while in the group without this arrhythmia it was 5%.

AF was a predictor of both in-hospital and late mortality in this study [38]. Rathore et al. showed, in a meta-analysis of 106780 elderly patients after AMI, that patients with AF had higher mortality rates compared to those without AF-intrahospital (23.3% vs. 16.0%); 3-day (29.3% vs. 19.1%) and 1-year (48.3% vs. 32.7%) [39]. Jabre et al. also published a meta-analysis that included 43 studies between 1970 and 2010 and concluded that AF is an independent predictor of mortality in ACS when other negative prognostic factors are excluded [40].

A retrospective cohort study involving 6705 patients with STEMI and NSTEMI showed that emerging AF in NSTEMI patients was an independent predictor of 30-day mortality while this was not the case for patients with STEMI. In both patient sub-populations, AF was associated with increased intrahospital complications [10]. Lopes et al. showed in their study the association of AF with both early and late mortality and in sub-populations of STEMI and NSTEMI patients [41]. In contrast to these studies, a study of 2475 patients with ACS by Kinjo et al. with emerging AF did not prove to be an independent predictor of intrahospital mortality, but was associated with an increased risk of intrahospital complications [19].

A recent multicenter retrospective cohort study examined STEMI patients from the proACSregistry (Portuguese Registry on Acute Coronary Syndromes) from 2010 and 2017. Patients who developed AF compared to those with sinus rhythm had more frequent intrahospital complications: higher prevalence of SI, cardiogenic shock, AV block, ventricular tachycardia, cardiac arrest, mechanical complications, stroke, and major bleeding, but AF was not shown as an independent predictor of mortality within STEMI [42].

Patients with AF have an increased risk of thromboembolic complications, especially ischemic stroke. The risk of ischemic stroke in patients with AF, according to ESC Guidelines, is estimated at about 5 times higher than in the general population, and the risk in these patients is not homogeneous but varies compared to many other factors. Common risk factors for ischemic stroke are summarized within the CHA2DS2-VASc score [1]. The GUSTO-I study registered a significantly higher incidence of ischemic stroke during the hospital treatment period of patients with newly developed AF and ACS compared to patients without this arrhythmia (3.1% vs. 1.3%, *p* < 0.01) [18].

In addition, the OPTIMAAL study showed that the newly developed AF had an independent predictive value for the development of ischemic stroke both in the first 30 days and in the follow-up period of 2.7 ± 0.9 years [5].

The controversial question remains whether the long-term clinical impact on the risk of unwanted clinical events of newly diagnosed AF in the acute phase of AMI differs from the impact of previously diagnosed AF before the onset of AMI. The results of the Obayashi Y study (6228 patients with AMI treated with percutaneous intervention, 7.9% with newly diagnosed AF, 9.5% with previously diagnosed AF and 82.7% without AF) showed that patients with newly diagnosed AF in AMI had a higher risk of mortality than those hospitalized due to heart failure and major bleeding compared with patients without AF and comparable risk as patients with previous AF. The risk of stroke was far higher in patients with newly diagnosed AF than in those with previously diagnosed AF [43]. This study may reinforce the concept that consideration of anticoagulant therapy is mandatory in patients with newly diagnosed AF in AMI who have a high risk of stroke (CHA2DS2-VASc score ≥ 2) even though the risk of bleeding is high.

## 7. Prevention of Thromboembolic Complications in AMI Complicated AF

About 6–8% of patients undergoing PCI have an indication for oral anticoagulant therapy with VKAs or NOACs [44]. The use of triple therapy in ACS is indicated in the case of patients with AF, mechanical valvulae and venous thromboembolism. The use of oral anticoagulant therapy should be consistent with individual risk of bleeding as well as ischemic risk. Both HAS-BLED and CHA2DS2VASc scores are most commonly used for risk assessment. When choosing VKAs or NOACs, it is important to determine if there is an associated valvular heart disease. In all patients with artificial heart valvulae as well as in patients with moderate or significant mitral stenosis, oral anticoagulant therapy is indicated regardless of other cardioembolic risk factors, and only VKAs drugs are recommended.

In all other patients with AF and one or more associated CHA2DS2VASc thromboembolic risk factors, the use of oral anticoagulant drugs is indicated, and NOACs are preferred. Compared with control or placebo, VKAs therapy reduces the risk of ischemic stroke by 64% and mortality by 24% [1]. Their use is limited by a narrow therapeutic interval, which requires frequent monitoring of the international normalized ratio (INR) and frequent dose adjustments. For optimal thromboprophylaxis, it is necessary that the TTR (time in therapeutic range) be > 70% [45]. In a comparison of efficiency in the prevention of thromboembolic complications, NOACs have been shown to be as efficient as VKAs [46,47,48,49].

These results were supported also by a meta-analysis showing that the use of NOACs reduces the risk of ischemic stroke and systemic embolism by 19% compared to the use of VKAs [50]. NOACs have been shown to be safer in relation to the risk of hemorrhagic stroke and other intracranial hemorrhages [1]. In the AUGUSTUS study, major or clinically relevant non-major bleeding was reported in 10.5% of patients receiving apixaban, compared with 14.7% of patients receiving VKA [51].

The lower mortality and the incidence of hospitalizations was in the group receiving apixaban compared to the group receiving VKA (23.5% vs. 27.4%), while the incidence of ischemic complications was similar.

Comparing the efficiency and occurrence of complications during dual antiplatelet therapy (NOAK + P2Y12 inhibitor) and triple antiplatelet therapy (VKA + P2Y12 inhibitor + Aspirin), results of PIONER AF-PCI studies with rivaroxaban, a RE-DUAL PCI study with dabigatran, AUGUSTUS with apixaban, and ENTRUST-AF PCI with edoxaban were very similar. Significant reductions in major or clinically significant bleeding have been registered in patients receiving NOAK + P2Y12 inhibitor therapy. Approximate rates of ischemic stroke and a neutral effect on clinically defined major adverse cardiovascular events and all causes of mortality were reported, comparing dual (NOAK + P2Y12 inhibitor) and triple therapy (VKA + P2Y12 inhibitor + Aspirin), (Table 1) [51,52,53,54].

In addition, the advantage of NOACs over VKAs is in the use of fixed doses, where there is no need for successive controls of INR, which increases the patient’s compliance in taking these drugs. When choosing a P2Y12 receptor blocker, the use of prasugrel or ticagrelor was associated with a higher risk of major bleeding compared to clopidogrel, and their use should be avoided in patients with ACS and AF [55,56]. Andreou et al. showed that the use of ticagrelor in double or triple antithrombotic therapy is associated with higher rates of clinically significant hemorrhagic complications compared to clopidogrel.

Moreover, the use of ticagrelor compared to clopidogrel in triple antithrombotic therapy has been associated with a higher incidence of MACE, while this was not the case when ticagrelor was used as part of dual antithrombotic therapy [56]. Dual antithrombotic therapy including OAC and P2Y12 receptor inhibitor has been associated with two to three times lower bleeding rates than triple therapy. However, available evidence suggests that shorter triple antiplatelet therapy is desirable to reduce thromboembolic complications in the first days after AMI [1].

According to the guiding recommendations, in case of acceptable hemorrhagic risk, triple therapy of NOACs with P2Y12 inhibitor as well as aspirin is recommended for the first week to one month, and after that it is necessary to continue NOACs with P2Y12 inhibitor until the expiration of 12 months since ACS, after which only NOACs are left in therapy.

**Figure 2:**
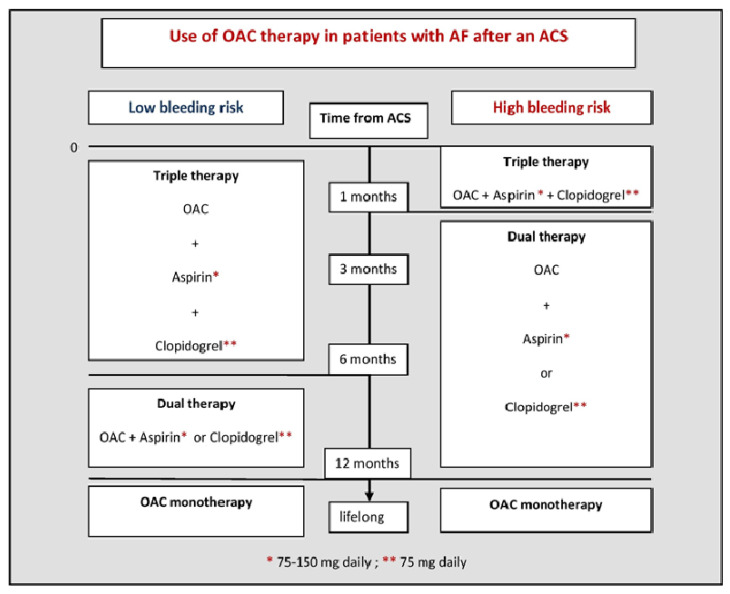
Use of OAC therapy in patients with AF after an ACS [57]. Legend: * Aspirin dose 75–150 mg daily; ** Clopidogrel dose 75 mg daily; ACS—acute coronary syndrome; AF—atrial fibrillation; OAC—oral anticoagulation (using vitamin K antagonist or non-vitamin K antagonist oral anticoagulants).

In patients with markedly increased ischemic risk, the duration of triple therapy should be extended to 3–6 months, while in cases of markedly increased hemorrhagic risk, dual therapy in combination with NOACs and P2Y12 inhibitor may be used from the outset. If there are no new ischemic events within 12 months of ACS/PCI, in the further course monotherapy with NOAC should be applied. Combining OAC therapy with P2Y12 receptor inhibitors such as ticagrelor and prasugrel is not currently recommended due to the increased risk of bleeding [57] (Figure 2).

Many studies have confirmed the positive effect of statins in the secondary prevention of ischemic stroke. The results of a study by Wankowicz et al. analyzed the effect of statin therapy before ischemic stroke on AF-related ischemic stroke in patients with well-controlled ventricular rate [58]. This retrospective multicenter analysis included 2309 patients with acute ischemic stroke. The results of the study showed that patients who used statins prehospitally had significantly lower neurological deficits compared with patients with stroke and AF who had not used statins before. Intrahospital mortality was also significantly higher in patients with atrial fibrillation-related stroke who did not take statins before hospitalization than in those who did. The results of this study suggest that the addition of statins to oral anticoagulants may be of great help in preventing stroke associated with atrial fibrillation, which must be confirmed by well-designed randomized, controlled studies.

## 8. Treatment of Patients with AF within AMI

The presence of AF within AMI has been shown to be associated with a higher rate of reinfarction, more frequent ischemic strokes, a higher incidence of heart failure, and a correlation with an increased risk of sudden cardiac death [59]. Therefore, adequate control of frequency, rhythm and the application of adequate prophylactic anticoagulant therapy is necessary. The RACE II study, conducted on the general population with AF, did not show that heart-rate control improves the quality of life or outcome in these patients [60]. In AMI, poor control of the rate of ventricular response during AF can worsen the symptoms of myocardial ischemia and precipitate heart failure worsening because any increase in frequency shortens the diastolic phase of the cardiac cycle and thus compromises perfusion through coronary blood vessels [1].

Danish researchers in the DIAMOND study showed that heart frequency is an independent prognostic factor for ten-year mortality in patients with AMI regardless of the presence of AF [61]. In acute conditions such as AMI, the associated factors that may affect the increase in frequency, such as anemia, infection, hypotension and hyperthyroidism, etc., should always be considered. In persons who do not have clinical symptoms and signs of heart failure as well as hypotension in AMI, intravenous beta-blockers are indicated for frequency control. Normotensive patients with extensive left ventricular damage and heart failure during AMI should receive amiodarone for heart frequency control, and digitalis may also be used in hypotensive patients [59].

Combined drug therapy may also be considered if monotherapy does not achieve the desired frequency. When using the combination of amiodarone and digoxin, it is necessary to monitor the concentration of digoxin in the blood due to the possible toxic effects of this drug. In the case of AF with a slow ventricular response, atropine may be used in hemodynamically stable patients, while in the case of symptomatic bradyarrhythmia, the option of choice is either emergency cardioversion or temporary pacemaker implantation [62]. Rhythm control in AF refers to attempts to establish and maintain sinus rhythm and in addition to the drug approach may include electrical cardioversion as well as catheter ablation.

The use of propafenone, vernakalant and flecainide is not recommended in patients with known coronary artery disease [1]. In the case of hemodynamic instability in patients with AF, urgent electrical cardioversion is necessary. Administration of amiodarone prior to electroconversion has been shown to improve the likelihood of sinus rhythm conversion in patients with AF [63]. In addition, a study by Schmidt et al. showed that the application of a maximum energy of 360 J is more effective in cardioversion of AF to sinus rhythm compared to a successively increased energy dose (125 J–150 J–360 J) [64].

During electrical cardioversion, it is necessary to sedate the patient, the use of benzodiazepines (midazolam or diazepam) is recommended, and the use of propofol is also possible [65]. During the procedure, it is necessary to monitor the patient’s blood pressure and saturation with pulse oximetry.

## 9. Risk Assessment and Prevention of Hemorrhagic Complications in Patients with AF and AMI

Provision of dual (i.e., an anticoagulant drug and one antiplatelet agent) or triple (i.e., an anticoagulant drug and two antiplatelet agents) antithrombotic therapy in patients with AF and AMI undergoing PCI procedure is associated with increased risk of hemorrhagic complications, especially in vulnerable individuals. These include patients of advanced age, severe renal or hepatic dysfunction, cancer, cognitive impairment or recent bleeding episode. Current guidelines recommend the assessment of bleeding risk with the use of a validated risk score, the HAS-BLED score before commencing the anticoagulant therapy [1]. Individuals identified to have a HAS-BLED score of three and above are considered to have high bleeding risk (>10% per year) and merit careful attention when deciding on the type of anticoagulant drug, the combination of antiplatelet agents and the duration of dual or triple antithrombotic therapy. Firstly, all modifiable bleeding risk factors (e.g., uncontrolled hypertension, labile INR, frequent use of non-steroidal anti-inflammatory drugs or alcohol abuse) should be addressed. In that respect, the use of NOACs instead of VKAs has been shown to reduce bleeding events (in particular, intracranial hemorrhage) and obviate hazards associated with labile INR. Secondly, the duration of triple therapy should be kept at minimum, balancing the risk of potential stent thrombosis against the risk of bleeding [66,67]. Available evidence suggests that the minimum duration of triple therapy should last seven days after stent implantation, and thereafter, individuals at high bleeding risk and low risk of stent thrombosis can safely continue with dual antithrombotic therapy, which confers a significantly lower risk of bleeding. In individuals at high risk of stent thrombosis (i.e., multivessel PCI, complex revascularization of left main coronary artery, coronary bifurcations, a history of stent thrombosis, diabetesand chronic kidney disease, etc.), the optimal duration of triple therapy should be a shared decision between the interventional cardiologist and the treating physician.

Bleeding risk is often dynamic and may increase in time due to the occurrence or worsening of concomitant illnesses (i.e., renal or hepatic dysfunction, stroke, bleeding) or introduction of new drugs. Therefore, attention should be given to the changes in the bleeding risk profile, in particular in vulnerable patients, since these changes strongly correlate with the escalating risk of hemorrhagic complications [68]. A randomized study has demonstrated that dynamic monitoring and reassessment using the HAS-BLED score in conjunction with the mitigation of modifiable risk factors were associated with a lower risk of major bleeding events compared to the usual care [69].

## 10. Conclusions

Despite the fact that modern methods of treating acute coronary syndrome (application of thrombolytic therapy and primary percutaneous intervention with stent implantation in infarcted arteries) provide rapid recanalization of infarcted blood vessel and thus less frequent arrhythmias, it is concluded that the incidence of atrial fibrillation is still high.

Adequate treatment of hypertension and diabetes is necessary in order to reduce the occurrence of atrial fibrillation. Given that different degrees of renal impairment, from albuminuria to severe renal failure, are associated with a higher incidence of AF, adequate rehydration of patients with acute coronary syndrome is necessary to prevent prerenal renal failure and reduce the incidence of contrast-induced nephropathy.

The occurrence of AF with rapid ventricular rhythm is associated with more frequent complications: development of heart failure, extension of infarction and increased risk of sudden cardiac death, and the use of anticoagulant therapy during hospitalization and later indefinitely is necessary, which reduces the risk of ischemic stroke and systemic embolization. However, since the use of triple and then dual antiplatelet therapy is associated with a risk of bleeding, frequent re-evaluation of the risk of both ischemia and bleeding is necessary.

In particular, risk/benefit assessment (risk scores) should be performed in patients taking VKAs due to variations in INR values. The use of NOACs has facilitated the treatment of patients with AF and ACS, and they are recommended as a treatment option, especially in patients who have no contraindications to their use. A lower rate of bleeding has also been reported when co-administered with P2Y12 inhibitors, in particular clopidogrel. The use of NOACs with more potent P2Y12 inhibitors (ticagrelor and prasugrel) should be avoided due to the risk of bleeding.

### Potential Studies Limitations

Most of the studies cited in this paper diagnosed AF on the basis of electrocardiograms and not on the basis of Holter electrocardiograms/continuous rhythm monitoring, which is why short-term AF (paroxysmal AF) could have been neglected. In addition, the occurrence of newly diagnosed AF in AMI might not have been registered.

## Figures and Tables

**Table 1:** Main characteristics and results of the PIONER AF-PCI, RE-DUAL PCI, AUGUSTUS and ENTRUST-AF PCI trials [1,51,52,53,54].

Clinical Trial	Year of Publication	Cohort Size (*n*)	Primary PCI	Randomization Window after Index Event	TAT Regimen Duration (Months)	Follow Up (Months)	Treatment Strategy	Safety Endpoint Major or CRNM ISTH Bleeding	MACE *
PIONEER AF-PCI [52]	2016	2124	38.5%	72 h	1, 6 or12	12	Rivaroxaban 15 mg + P2Y12	16.8%	6.5%
							Rivaroxaban 2.5 mg bid + P2Y12 + aspirin	18%	5.6%
							VKA + P2Y12 + aspirin	26.7%	6%
RE-DUAL PCI [53]	2017	2725	50.5%	120 h	1 (BMS) or 3 (DES)	14	Dabigatran 110 mg bid +P2Y12	15.4%	15.2%
							Dabigatran 150 mg bid + P2Y12	20.2%	11.8%
							VKA + P2Y12 + aspirin	26.9%	13.4%
AUGUSTUS [51]	2019	4614	37.3%	14 days	6	6	Apixaban 5 mg bid+ P2Y12 + aspirin	10.5%	6.7%
							Apixaban 5 mg bid + P2Y12		
							VKA+ P2Y12+ aspirin	14.7%	7.1%
							VKA+ P2Y12		
ENTRUST-AF PCI [54]	2019	1506	52%	5 days	1–12	12	Edoxaban (60 mg) + P2Y12	17%	7%
							VKA +P2Y12 + aspirin	20.1%	6%

Legend: BMS = bare-metal stent; CRNM = clinically relevant non-major; DES = drug-eluting stent; ISTH = International Society on Thrombosis and Haemostasis; PCI = percutaneous coronary intervention; TAT = triple antithrombotic therapy; VKA = vitamin K antagonist; MACE * = major adverse cardiac event.

## Data Availability

Not applicable.
